# Laser-guided real-time automatic target identification for endoscopic stone lithotripsy: a two-arm in vivo porcine comparison study

**DOI:** 10.1007/s00345-020-03452-0

**Published:** 2020-09-22

**Authors:** Daniel Schlager, Antonia Schulte, Jan Schütz, Albrecht Brandenburg, Christoph Schell, Samir Lamrini, Markus Vogel, Heinrich-Otto Teichmann, Arkadiusz Miernik

**Affiliations:** 1 Department of Urology, Faculty of Medicine, Medical Center – University of Freiburg, Hugstetter Str. 55, 79106 Freiburg, Germany; 2 Fraunhofer Institute for Physical Measurement Techniques IPM, Heidenhofstrasse 8, 79110 Freiburg, Germany; 3 Institute of Surgical Pathology, Faculty of Medicine, Medical Center – University of Freiburg, Hugstetter Str. 55, 79106 Freiburg, Germany; 4 LISA Laser Products GmbH, Albert-Einstein-Straße 4, 37191 Katlenburg-Lindau, Germany

**Keywords:** Feedback control, Autofluorescence, Holmium laser, Laser lithotripsy

## Abstract

**Introduction and objective:**

Thermal injuries associated with Holmium laser lithotripsy of the urinary tract are an underestimated problem in stone therapy. Surgical precision relies exclusively on visual target identification when applying laser energy for stone disintegration. This study evaluates a laser system that enables target identification automatically during bladder stone lithotripsy, URS, and PCNL in a porcine animal model.

**Methods:**

Holmium laser lithotripsy was performed on two domestic pigs by an experienced endourology surgeon in vivo. Human stone fragments (4–6 mm) were inserted in both ureters, renal pelvises, and bladders. Ho:YAG laser lithotripsy was conducted as a two-arm comparison study, evaluating the target identification system against common lithotripsy. We assessed the ureters’ lesions according to PULS and the other locations descriptively. *Post*-*mortem* nephroureterectomy and cystectomy specimens were examined by a pathologist.

**Results:**

The sufficient disintegration of stone samples was achieved in both setups. Endoscopic examination revealed numerous lesions in the urinary tract after the commercial Holmium laser system. The extent of lesions with the feedback system was semi-quantitatively and qualitatively lower. The energy applied was significantly less, with a mean reduction of more than 30% (URS 27.1%, PCNL 52.2%, bladder stone lithotripsy 17.1%). Pathology examination revealed only superficial lesions in both animals. There was no evidence of organ perforation in either study arm.

**Conclusions:**

Our study provides proof-of-concept for a laser system enabling automatic real-time target identification during lithotripsy on human urinary stones. Further studies in humans are necessary, and to objectively quantify this new system’s advantages, investigations involving a large number of cases are mandatory.

**Electronic supplementary material:**

The online version of this article (10.1007/s00345-020-03452-0) contains supplementary material, which is available to authorized users.

## Introduction

Photonic technology has evolved as an essential technology for treating urinary stones. Laser lithotripsy is now considered one of the most important treatment techniques by which to disintegrate urinary stones for patients undergoing semirigid ureteroscopy (URS) or percutaneous (PCNL) stone removal [1, 2]. Over the past 2 decades, the pulsed Holium:YAG laser (Ho:YAG) has become the lithotrite of choice [3, 4]. The Holmium laser produces light with a wavelength of 2100 nm and uses a mainly photothermal mechanism to fragment calculi [5]. As this wavelength is so strongly absorbed by water, it can damage the surrounding tissue. While severe direct physical lesions occur seldom, there is increasing evidence that secondary thermal injuries due to excessive temperatures in the upper urinary tract can become dangerous [6–8]. The Ho:YAG laser’s therapeutic range in aqueous liquid is about 1–5 mm, and the release of high energetic pulses depends solely on the surgeon. Our research group recently managed to deliver solid in vitro evidence that monitoring the autofluorescence spectra of calculi allows precise target differentiation between stone, tissue, and endoscope components [9]. We developed an optical feedback module utilizing the autofluorescence collected from the objects in front of the laser fiber tip for the purpose of releasing laser emission in the presence of autofluorescence solely from a stone, or to inhibit laser emission in the absence of autofluoresence. Pulse emissions were controlled exclusively by our new target system, and impulses were only released when stone material was detected within the laser fiber’s therapeutic range. We hereby present our results from an in vivo porcine animal model. We believe that inhibiting the laser in the absence of stone autofluorescence will reduce the risk of laser-induced urothelial damage to a minimum and decrease the total energy release into the upper urinary tract, thus enabling better temperature management.

## Materials and methods

### Study design

This study was approved by the committee on animal experimentation of the regional council (registration no: 35-9185.81/G-16/169), and the local university medical center ethics committee (IRB no: 296/15). This study was performed in strict accordance with the “Protection of Animals Used for Scientific Purposes” act (2010/63/EU).

A standard Ho:YAG laser lithotripsy was performed comparing our group’s newly designed and experimentally built laser target recognition system, which integrates a spectroscopic autofluorescence feedback system enabling real-time analysis [10] of the target, to standard Ho:YAG laser lithotripsy without target recognition system. Surgery was performed in vivo by an experienced endourology team. Two female domestic pigs (66/74 kg BW) underwent semirigid URS, PCNL, and bladder stone lithotripsy under general anesthesia. The following procedures were carried out: URS with the new target system turned off (functionality of a standard commercially available Ho:YAG laser), URS with the new target system assisting the surgeon on the contralateral kidney. The same approach was taken performing PCNL on the second animal. We additionally performed bladder stone lithotripsy with the new target system activated in the first pig and inactivated in the second one.

**Fig. 1:**
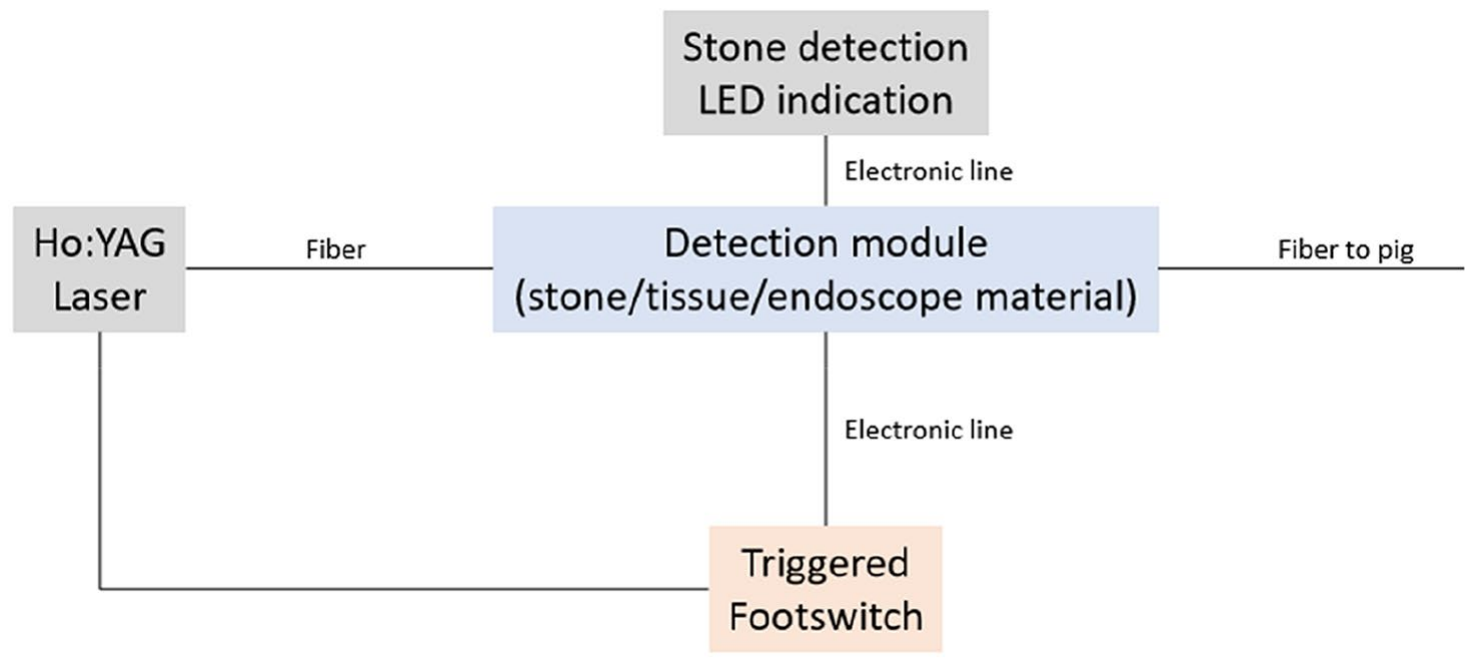
Schematic arrangement of the functional module of the spectral feedback system used in this study

Due to the broad spectrum of the wavelengths we applied, the optical setup was demanding (fluorescence excitation 520 nm, fluorescence 550–750 nm, and laser 2100 nm). A light beam coupler was, therefore, used for signal transmission into the therapy fiber. The target system was coupled to the laser footswitch activation system and was triggered by the endourology surgeon. The schematic arrangement of the functional module of the spectral feedback system used in this study is shown in Fig. 1. Laser energy emission was only released when stone material was detected by the target system.

### URS with inactivated/activated target system

Prior to the procedure, human calcium oxalate stones with a standardized stone weight of 100 mg in total were placed retrograde into the kidneys and ureter using a flexible ureteroscope (Cobra^®^, Richard Wolf GmbH, Knittlingen, Germany) and nitinol grasper (Ngage^®^, Cook Medical, Bloomington, IN/USA). During semirigid URS (semirigid scope 7.5/9.8 Fr., Richard Wolf GmbH, Knittlingen, Germany), we performed lithotripsy using a Holmium laser (Sphinx jr.™, LISA Laser Products GmbH, Katlenburg-Lindau, Germany) with high frequency (20 Hz) and low energy (0.5 J). During the procedure, the new target system was active for the right kidney and inactive for the left kidney.

### PCNL with inactivated/activated new target system

We employed ultrasound-guided access to the dilated collecting system. The tracts were dilated using single-use dilators 6–20 F (Cook Medical, Bloomington, IN/USA). An 18F nephroscope (Richard Wolf GmbH, Knittlingen, Germany) was inserted. Similar to URS, a stone material of 200 mg in total was placed into the renal pelvis using a grasper. High frequency (15 Hz) and low energy (1 J) were used as the laser setting for lithotripsy.

### Bladder stone lithotripsy with inactivated/activated new target system

For lithotripsy in the bladder, calcium oxalate stones with a standardized stone weight of 450 mg in total were placed retrograde into the bladder using a flexible ureteroscope (Cobra^®^, Richard Wolf GmbH, Knittlingen, Germany) and nitinol grasper (Ngage^®^, Cook Medical, Bloomington, IN/USA). During cystoscopy, we performed lithotripsy using a Holmium laser (Sphinx jr.™, LISA Laser Products GmbH, Katlenburg-Lindau, Germany). Settings were: frequency (20 Hz) and energy output (1.0 J). This new target system was active for the first and inactive for the second bladder during the procedure.

During the trial, we measured the amount of energy (J) emitted by the laser during the respective treatment with the same amount of uroliths (g).

The urinary tract was evaluated for the presence of laser-induced lesions after completing the procedure according to the standards applied in human surgery (PULS classification at the ureter, qualitative, and quantitative descriptive assessment of lesions in the renal pelvis and bladder, and retrograde examination with contrast medium) [11].

Immediately after the termination of surgery, the anesthetized pigs were euthanized via an intravenous application of potassium chloride. Immediately after transperitoneal cysto-uretero-nephrectomy, kidney–ureter–bladder specimens were subjected to pathological macroscopic and microscopic assessment.

**Fig. 2:**
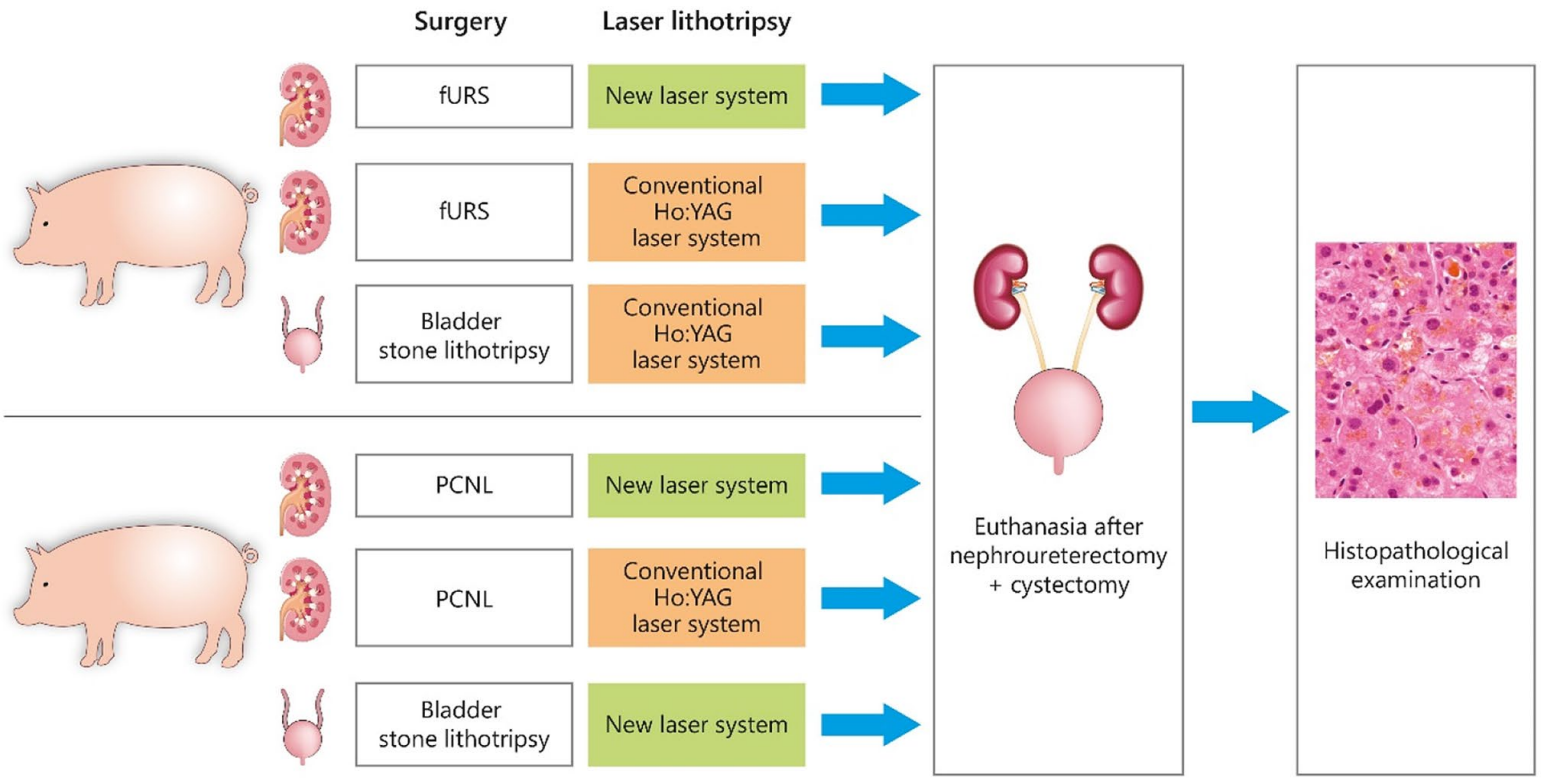
Study design and workflow (green—study arm, orange—control group)

Our standard Ho:YAG laser lithotripsy (spectral feedback off) results were compared to the results which we had obtained with the new target system assisting the surgeon (spectral feedback on) as Fig. 2 indicates.

### Statistical analysis

Descriptive statistical calculations were performed using Microsoft Excel 2010 (Microsoft Corporation, Redmond, WA, USA).

## Results

Lithotripsy of the inserted stone material succeeded with both endoscopic systems (spectral feedback on/off).

After the completion of treatment, multiple superficial and deep thermal injuries to the urothelium were revealed macroscopically via endoscopic examination on site. These findings were only partially confirmed on histopathological assessment.

**Fig. 3:**
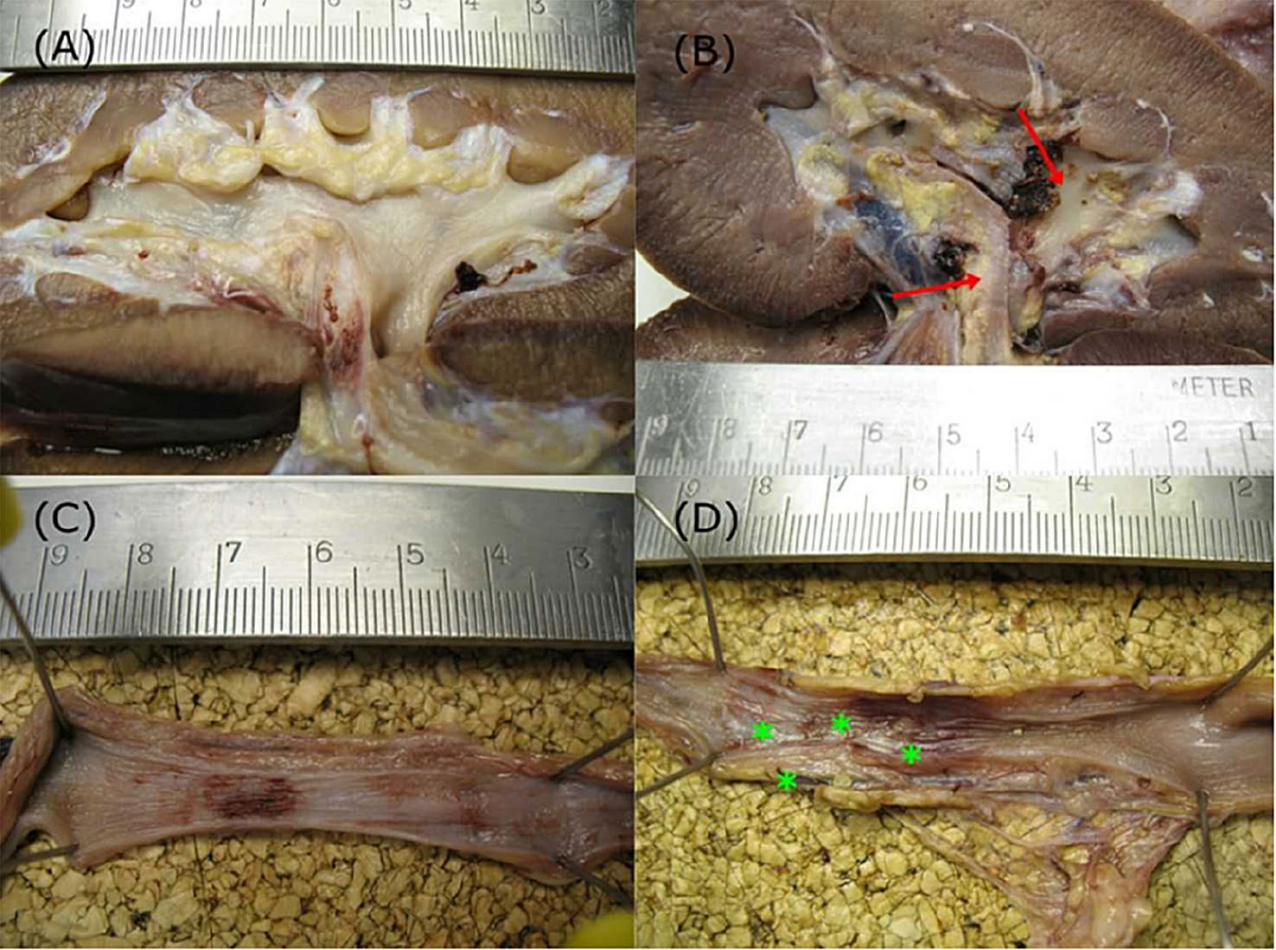
**a** Macroscopic images of a *post mortem* renal pelvis after an endoscopic laser lithotripsy during PCNL using our new laser system, **b**macroscopic images of a *post mortem* renal pelvis after an endoscopic laser lithotripsy during PCNL using a standard holmium laser (red arrows—extensive mucosa lesions), **c** macroscopic images of a *post mortem* ureter after endoscopic laser lithotripsy during semirigid URS using the new laser system, **d** macroscopic images of a *post mortem* ureter after endoscopic laser lithotripsy during semirigid URS using a standard holmium laser (green asteriks—multilocular thermal superficial mucosa lesions)

In comparison, we detected no macroscopic damage to the urothelium after treatment with the new target system (according to the PULS classification and qualitative description relying on visual appearance). Moreover, microscopic and histological examinations revealed no relevant thermal damage (Fig. 3). There was no evidence of any organ perforation either with the feedback off or on during fluoroscopic and macroscopic examination. Table 1 illustrates both semi-quantitative and qualitative findings of these examinations. To visualize the differences between the standard system and our new target system, a video of the procedure is provided in the supplementary material.

**Table 1:** Semi-quantitative and qualitative results of postinterventional assessment

Animal	Localisation	Procedure	Laser system	Quantitative examination (PULS 0-5)	Qualitative Examination (by surgeon)
1	Right kidney	PCNL	New target system	n.a.	Isolated barely visible minor lesions
1	Left kidney	PCNL	Conventional Ho:YAG	n.a.	extensive superficial lesions
1	Bladder	Stone lithotripsy	New target system	n.a.	Abrasions of mucosa by urinary stone, no thermal lesions
2	Right ureter	URS	New target system	0	No clinically relevant lesions
2	Left ureter	URS	Conventional Ho:YAG	2	Extensive superficial lesions
2	Bladder	Stone lithotripsy	Conventional Ho:YAG	n.a.	Extensive superficial lesions

*PULS* postureteroscopic lesion scale, *n.a.* not applicable

Furthermore, we observed that by having the same urinary stone mass (g) in each treatment, the energy (J) the laser emitted was less with the target system switched on than when applying the standard method: for semirigid URS using a commercially available holmium laser, 5322 J were required, compared to 3882 J using the new target system. The emitted energy during PCNL diverged even further: 8055 J for the standard holmium laser and 3854 J for the new target system. We noted the same tendency during bladder lithotripsy, as 3267 J were applied with the standard method and 2707 J with our new target system.

Table 2 summarizes the trial settings for each procedure.

**Table 2:** Summary of settings

Animal	Localization	Procedure	Laser system	Energy emitted (J)	Stone weight (mg)	Setting/pulse rate
1	Right kidney	PCNL	New target system	3854	200	15 Hz/0,5 J
1	Left kidney	PCNL	Conventional Ho:YAG	8055	200	15 Hz/0,5 J
1	Bladder	Stone lithotripsy	New target system	2707	450	20 Hz/1,0 J
2	Right ureter	URS	New target system	3882	100	20 Hz/1,0 J
2	Left ureter	URS	Conventional Ho:YAG	5322	100	20 Hz/1,0 J
2	Bladder	Stone lithotripsy	Conventional Ho:YAG	3267	450	20 Hz/1,0 J

**Fig. 4:**
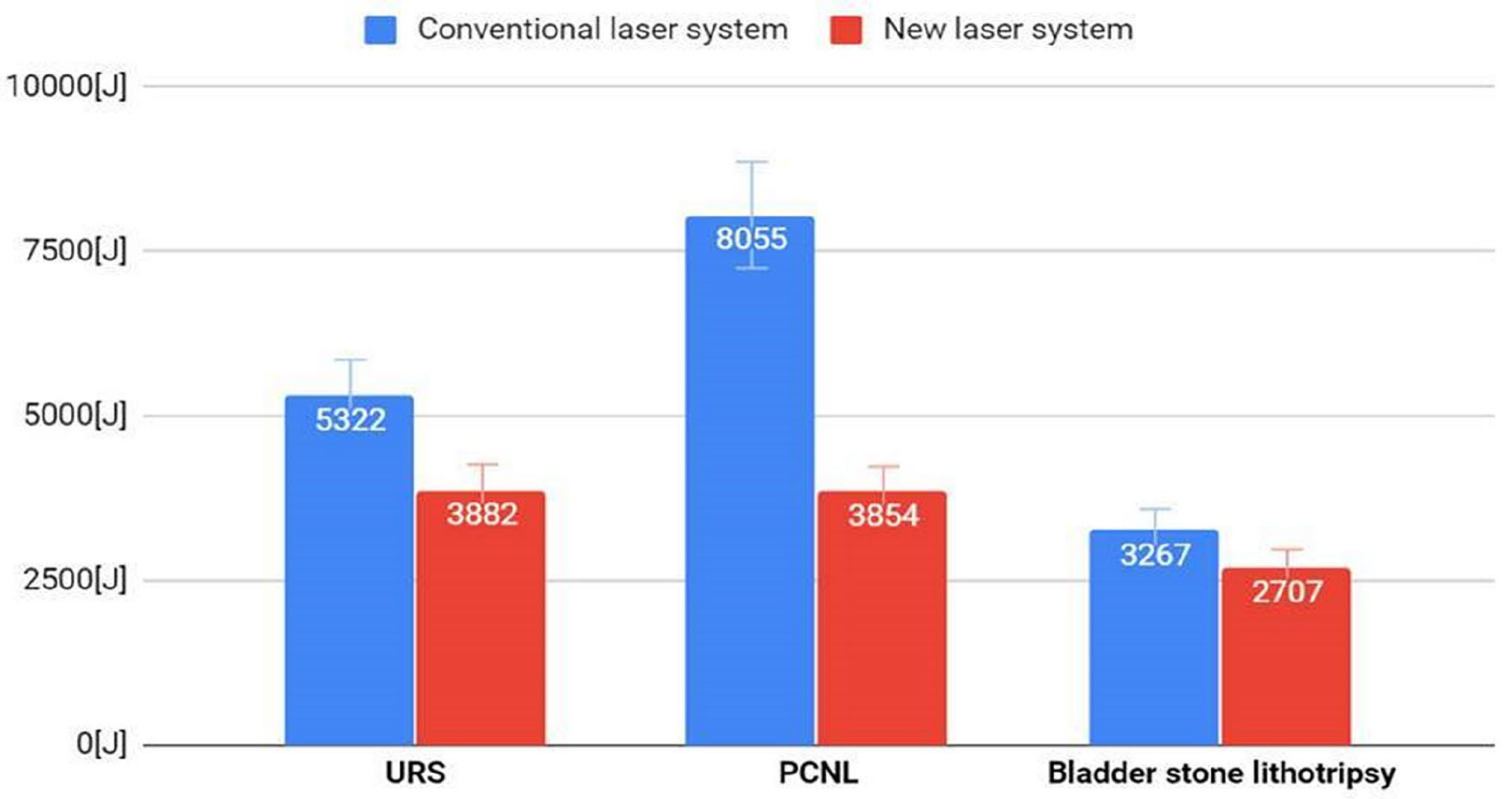
Comparison of total energy emitted for standard and new target system

Figure 4 shows each procedure’s total emitted energy comparing the standard laser to our new system with the target recognition activated. One important parameter is the total energy emitted during the procedures, since it is directly related to the heat induced within the urinary tract.

We investigated this parameter during each of the three procedures involving activated and inactivated target recognition systems.

The applied energy was significantly less, with a mean over 30% reduction in energy emitted (URS 27.1% less, PCNL 52.2% less, and bladder stone lithotripsy 17.1% less).

In summary, we observed reduced laser energy emission during each procedure when using our target recognition system.

From the user’s point of view, this new target system also offers good functionality and handling, so that the procedures were carried out without any noteworthy incidents.

## Discussion

We conducted an in vivo porcine model study for intraoperative analysis using autofluorescence that only allows the laser to emit energy pulses when the stone material is within immediate therapeutic reach of the laser fiber tip, while preventing laser impulses when the fiber tip is aiming at urothelial lining, the endoscope itself or when there is no target at all.

To prevent tissue injury, we carried out real-time autofluorescence-differentiation of the substance that the laser is currently irradiating. This principle is based on the fact that kidney stones emit characteristic autofluorescence radiation when excited at the short wavelength range of visible light (e.g., 520 nm), which differs from that expected in human body tissue [9]. The laser was connected to the feedback module by a fiber optic cable. The laser was enabled or blocked by means of a modified foot switch which received a digital signal from the demonstrator system. In this system, the green laser light triggering fluorescence excitation was superimposed on the laser. Both beams were coupled within the same fiber. The autofluorescence is partly collected by the fiber, spectrally separated from the remaining light for analysis, and guided to a detector. Due to the wide spectrum of wavelengths involved, the optical setup was very demanding (fluorescence excitation 520 nm, fluorescence 550–750 nm, and laser 2100 nm). A fiber coupler was used for coupling into the application fiber.

Lange et al. [12] obtained similar findings regarding the usefulness of autofluorescence for stone detection in an in vitro study. However, we took it one step further: This innovative technology was now—after having completed in vitro studies—successfully tested in large animal models, and extensive preclinical data were collected [10]. This proof-of-concept study shows that, during simulated kidney stone fragmentation procedures in domestic pigs, under conditions—that closely match human patients’ treatment—kidney stones, tissue, and endoscope components can be correctly differentiated in real time. The results of our postoperative, standardized tissue-damage assessment (according PULS classification) demonstrated that injuries can be reduced by inhibiting laser emission on urinary tract tissue through our new target system using real-time spectral information to differentiate between genuine stone material and its absence. Pathological examinations consequently substantiated these findings by revealing that no relevant lesions occurred through either procedure.

The main advantage of our new target system is significantly reduced energy output to achieve the same therapeutic goal, namely, the sufficient disintegration of a urinary calculus. Fewer lesions were endoscopically visible in the test animals’ urinary tracts. These did not appear to have been caused by the laser itself, but rather by micromovements and friction from the stone during the lithotripsy procedure (microhematomas in the muscosa). However, these lesions affected only superficial urothelial layers and did not result in manifest organ perforations. A long-term functional and morphological effect of these lesions on the urinary tract cannot be demonstrated through an experimental setting entailing postoperative euthanization of experimental animals and immediate preservation of the preparations.

Pathological examination substantiated our visual assessment of the urinary tracts via endoscopy and fluoroscopy with contrast dye. While the pathological evaluation of these two experimental animals detected no major, significant differences between the different treatment modalities, this is expected due to our study design and low number of observations. Further studies will be required to substantiate this macroscopic evaluation based on the initial observations.

Furthermore, this type of technology can allow safer lithotripsy when due to kidney anatomy, the stone is not visible. The fiber can be inserted and this fail-safe system would allow to disintegrate these otherwise difficult to reach calculi in a safe manner.

Additionally, we believe that operative time can be significantly reduced. Since less-experienced surgeons might be more hesitant to release laser energy more continuously, work flow decreases and this technology would surely reduce operative time.

This new target system also paves the way as a safety mechanism for future robotic and automated laser lithotripsy systems, because it can be used as one of the fail-safe mechanisms preventing tissue damage.

In summary, we propose that a favorable risk/benefit assessment can be derived, as our results clearly indicate that the safety of endoscopic stone interventions could improve substantially by causing less damage to surrounding tissue while preventing severe thermal damage to endoscopes during Ho:YAG lithotripsy. It is also very likely that human patients will benefit equally thanks to this system’s functionality, which needs to be proven in imminent further investigations. We have demonstrated that our newly developed target system reduces the maximum of the energy delivered, indicating that this procedure’s invasiveness might also have been significantly minimized. As an advanced safety feature, this new target system may also enhance patient safety by benefiting significantly less-experienced surgeons undergoing training [13]. Our new target system with its feedback mechanism provides the user with the certainty that even if the laser is fired unintentionally, no laser emission will occur, and therefore, no tissue damage and no unnecessary heat build-up are likely. The same mechanism is also probably compatible with any other laser (e.g., Thulium laser), but this will have to be proven in future studies [14, 15]. With this intelligent laser technology, new possibilities and an entirely new approach in the field of medical interventions are created.

Our study has certain limitations. Obviously, it was not possible to measure temperature during the operation on site. The primary idea of the authors was to imitate real conditions in animal models as closely as possible. Introducing measurement sensors could have led to changes in conditions with complications such as bleeding or injury to the urinary tract. We, therefore, decided against using them. Furthermore, it needs to be underlined that it remains unclear whether the system can provide the same advantages when using other techniques like the fragmentation technique, as this was not part of our study. There is a lack of clinical data by which to objectify the long-term effects of different temperatures on human urothelium [16]. It is, therefore, still unclear what real advantages this new target system will have regarding the minimization of the thermal effects of the laser.

As the next step, we are planning the first trial in patients. For reasons of cost and study design, this will involve a cohort of a maximum of 20 people. In the long term, of course, larger patient cohorts and long follow-up periods are necessary to discuss the effect of reduced and controlled laser energy output during stone fragmentation in a proper context.

## Electronic supplementary material

Below is the link to the electronic supplementary material.

Supplementary material 1 (MP4 52568 kb)

## Abbreviations

µm: Micrometer
arb. u.: Arbitrary units
BW: Body weight
e.g.: Exempli gratia, for example
g: Gram
Ho:YAG: Holmium:yttrium–aluminum–garnet
Hz: Hertz
J: Joule
mm: Millimeter
n.a.: Not applicable
nm: Nanometer
PCNL: Percutaneous nephrolithotripsy
PULS classification: Postureteroscopic lesion scale
URS: Ureteroscopy
